# California State Veterinary Medical Association

**Published:** 1892-02

**Authors:** R. A. Archibald

**Affiliations:** Secretary


### SOCIETY PROCEEDINGS.

*California State Veterinary Medical Association.*—The fourth annual meeting of the California State Veterinary Medical Association was held at the Baldwin Hotel, San Francisco, on Wednesday, December 9th, 1891. The meeting was called to order by the President, Dr. Thomas Maclay, of Petaluma, and upon roll-call the following gentlemen answered to their names, viz.: Dr. H. G. Pierce, of Ventura; Dr. J. K. Witherspoon, of Los Angeles; Dr. C. B. Orvis, of Stockton; Dr. D. F. Fox, of Salinas; Dr. H. F. Spencer, of San Jose; Dr. C. Masoero, and Dr. W. F. Egan, of San Francisco; Dr. W. E. Wadams, of Santa Clara; Dr. A. M. McCollum and Dr. R. A. Archibald, of Sacramento.

The minutes of the previous meeting were read and approved. Letters of regret for non-attendance were read from Drs. Davenport, Rowland, Blackington, Whittlesey, Oliver, Spencer, Sr., and Morrison. The President then made a few remarks in relation to the death of one of the members, Dr. W. H. W. Woodruff, of San Francisco. A committee was appointed to draft resolutions of respect to the memory of Dr. Woodruff, a copy of which was ordered to be endorsed and sent to his wife. Reports from the Secretary showed the Association to be in a flourishing condition both numerically and financially.

The next order of business being the election of officers for the ensuing year. The President declared nominations in order, and the same being declared closed, a ballot was then taken with the following result: Dr. W. E. D. Morrison, Los Angeles, President; Dr. W. F. Egan, San Francisco, Vice-President; Dr. R. A. Archibald, Sacramento, Secretary; Dr. I. B. Orvis, Stockton, Treasurer. The directors appointed were Drs. Spencer, Wadams and Burns. Drs. Maclay, Orvis, Masoero, Rowland and Whittlesey were appointed as a Board of Examiners. On motion the meeting adjourned until 10.30 A. M., Thursday, December 10th, 1891.

The meeting was resumed on Thursday at 10.30 A. M. The Vice-President, Dr. Egan, in the chair. Dr. C. B. Orvis, of Stockton, then read a valuable paper on reports of cases which had occurred in his practice. He also read an extract from the JOURNAL OF MEDICINE AND VETERINARY ARCHIVES, on Meningitis. A valuable discussion followed by all the members present.

Dr. H. F. Spencer, of San Jose, read a paper on Corn Stalk Disease. During the discussion that followed, Drs. McCollum, Maclay and Wadams said that they had seen this disease or something similar among cattle which were turned out in a corn-field after hogs had been in it and broken it down. Dr. Pierce, of Ventura, said that a sheepman near Ventura had lost some 30,000 head of sheep in the last two years from some disease unknown to him. He did not get a chance to make any post-mortem examination. A very instructive paper was then read by Dr. D. F. Fox, Salinas, on Quittor. The essayist described the different forms of Quittor minutely. He defined Quittor as a disease affecting the digital region of the horse, mule, ass, and frequently the bovine race. It may attack any class of horses, but the coarse-bred, thick-skinned, hairy-legged animals are the most frequently afflicted.

It presents different pathognomonic symptoms dependent upon the difference in its origin, progress and principal seat of evil, whether it be in the vascular, cellular tissues component of, or connected with the skin, the tendinous aponeurotic or ligamentous expansion of the cartilages and cartilage fibrous masses or the bones themselves. It is from a consideration of these structural differences that Quittor is divided into several classes, namely : Cutaneous or Simple, Tendinous, Sub. Horny, Cartilagineous Quittor.

In cattle there is more pain manifest than in horses, and the swelling always ex-. tends to the knee, rumination ceases and the animal evinces the greatest anguish

The slough is followed by a wound of varying depth, which sometimes exposes the diseased articular surface of the phalanges ; if this continues long the pus may affect the interdigital ligament, complications that render the case incurable.

An animated discussion followed, in which all the members joined. The following named gentleman was proposed for membership by Dr. McCollum, viz. : Dr. Davidson, of San Bernardino. On motion a vote of thanks was tendered to the essayists for the able manner in which they had entertained the meeting. Drs. Egan McCollum and Archibald were appointed as essayists for the next meeting.

On the transaction of some other business the meeting adjourned until the next quarterly meeting at the Baldwin Hotel, San Francisco, the 9th day of March, 1892. The next annual meeting to be held in Los Angeles, December 14th, 1892.
After the session the members and a few guests enjoyed a banquet at the Maison Riche. The banquet hall was most tastefully and appropriately decorated. Dr. W. F. Egan officiated as toastmaster. The menu was most elaborate and costly. The toast, "Our Profession," was responded to by Dr. Maclay, who gave a brief resume of the work of the past, and then pointed out in glowing terms the work of to-day in disseminating knowledge by this once despised profession, and how much good is being done to alleviate the sufferings of man's best friend. He spoke of the advancement made in the treatment of diseases that were at one time deemed incurable, and the enlightenment of all people regarding the spread of disease through the efforts of the professors of scientific veterinary practice. The position held by the Veterinarian to-day as sanaitarians in the world, and how powerful they will yet become from the beginning they have made in disclosing the spread of disease through the bacilli found in the lacteal fluid of diseased bovines was referred to, and his comparisons between the microbes in diseased animals and that found in tuberculosis in the human race were very instructive. His advice as to what the profession should do to call the attention of the municipal authorities to the fact that the meat inspectors appointed to condemn diseased meats are not Veterinarians ; his explanation of the strugglss the profession have had in Sacramento to get stringent laws passed to protect the Veterinarian by examination, the same as is now done to protect lawyers, physicians and pharmacists. The appointment of a State Venterinarian whose duty shall be to see that all laws for the destruction of horses and cattle afflicted with contagious diseases should be faithfully carried out, concluded his remarks. Our county members was feelingly responded to by Dr. C. B. Orvis. The press was the subject of a splendid response by Mr. M. C. Allen, who spoke of the efforts of the press to sustain the veterinarians in their legislative struggle for their rights, and the indebtedness humanity owes to this growing profession for its efforts to ameliorate the sufferings of the brute creation that cannot express their feelings by gift of speech, but by their muteness appeal to the tenderest sympathies of all mankind. Mr. Allen gave a clear and concise view of the stand the society had taken, in which the leading journals of the world were heartily in accord. Our young members was responded to by Dr. H. F. Spencer, and the promise of a greater interest and more faithful attendance to the needs of the Association by the young members was made. Our Legislative workers found an able friend and interpreter in Dr. McCollum, who revived the struggle the society has had in the past to receive protection. In a few words he explained how the society will yet achieve success. Veterinary practices of early days was explained in a very humorous manner by Captain Thos. B. Merry. Drs. Masoero, Fox. Archibald, Wadams, Pierce, Witherspoon and Egan also responded to the calls of those present.

Mr. Robert Mitchell, aided by Mr. F. H. Belcher and Dr Maclay, sang songs, duets and trios and gave recitations that were enjoyed by those present. Amusing stories were told and expressions of good will were heard from Messrs. Storn, Callundan and Culver. To the soul-stirring strains of that good song of fraternity, " Auld Lang Syne," the members departed from the hall where cares and troubles had been forgotten amid the joys of true companionship.

R. A. ARCHIBALD, *Secretary.*

